# An updated checklist of the vascular flora native to the State of Palestine – West Bank

**DOI:** 10.3897/BDJ.14.e188380

**Published:** 2026-03-31

**Authors:** Mohammed Saleem Ali-shtayeh, Salam Yousef Abuzaitoun, Rana Majed Jamous

**Affiliations:** 1 Biodiversity & Environmental Research Center, BERC, Nablus, Palestine Biodiversity & Environmental Research Center, BERC Nablus Palestine

**Keywords:** eastern Mediterranean flora, floristic checklist, plant endemism, phytogeography, IUCN Red List, conservation botany

## Abstract

The Palestinian West Bank is located within the eastern Mediterranean biodiversity hotspot and hosts a highly diverse native vascular flora despite its limited geographic extent and pronounced environmental gradients. In this study, we present a substantially updated checklist of the native vascular plants of the Palestinian West Bank, based on critical taxonomic revision, harmonised nomenclature and comprehensive synthesis of recent floristic evidence. The update integrates newly-published distributional records, digital herbarium and biodiversity database information, refined taxonomic circumscription using current international standards and recently completed national IUCN conservation assessments. The checklist comprises 1710 taxa (1668 species and 42 subspecies) belonging to 652 genera and 102 families, including 1695 angiosperms, five gymnosperms and ten ferns. The floristic composition is dominated by Fabaceae, Asteraceae, Poaceae, Caryophyllaceae, Brassicaceae and Lamiaceae, reflecting Mediterranean and Irano-Turanian chorotypes. A total of 151 taxa are endemic or sub-endemic to Palestine and adjacent regions. The IUCN assessments indicate that 28.2% of the native flora is classified as threatened. Remarkably, 29 taxa are considered extinct or possibly extinct at the national level, highlighting ongoing habitat degradation and land-use pressures. The checklist integrates phytogeographical distribution, life forms, chorotypes, habitat preferences and conservation status, providing a robust taxonomic and biogeographical baseline. This updated inventory represents a critical reference for floristic research, biodiversity monitoring and conservation planning in the Palestinian West Bank and the wider Levant.

## Introduction

A precise and continuously updated plant checklist is an essential foundation for plant systematics, biogeographical research and conservation planning, particularly in regions of high biodiversity and environmental heterogeneity. The West Bank of the State of Palestine is located at the convergence of four main phytogeographical regions, including the Mediterranean, Irano-Turanian, Saharo-Arabian and Sudanian, which creates a pronounced environmental gradient over a relatively small geographical area and supports a corresponding high diversity of native vascular plants. This floristic heterogeneity, further shaped by a long history of intensive human land use, positions the region as both an important centre of plant diversity and a landscape highly vulnerable to biodiversity loss.

The most comprehensive recent checklist of the vascular flora of the State of Palestine was published by Ali-Shtayeh and Jamous (Ali-Shtayeh and Jamous 2018), documenting 1,938 taxa for the West Bank and Gaza Strip. Subsequent re-assessments and taxonomic harmonisation led to revisions of this total, most notably through the annotated checklist of native vascular plants of the State of Palestine, which refined species circumscriptions, clarified native versus alien status and aligned nomenclature with international taxonomic standards (Ali-Shtayeh et al. 2022). In that checklist, a total of 1,826 native taxa were reported for Palestine as a whole, including 1,683 taxa recorded in the West Bank and 1,217 taxa documented in the Gaza Strip, of which 140 taxa were reported exclusively from Gaza. These developments reflect a broader Mediterranean trend towards dynamic, periodically revised national floras, facilitated by the adoption of unified taxonomic backbones and stricter inclusion criteria, as demonstrated by recent checklist updates for Italy, Cyprus and Greece (Hand et al. 2011; Dimopoulos et al. 2016; Bartolucci et al. 2024).

During the past decade, floristic knowledge of the Palestinian West Bank has expanded substantially through new distributional records, taxonomic revisions and conservation assessments published in peer‑reviewed journals, regional floras and institutional reports, particularly those produced by the Biodiversity and Environmental Research Center (BERC) and collaborating universities (Al-Sheikh and Mahassneh 2016, Ali-Shtayeh and Jamous 2018, Pahl and Qumsiyeh 2021, Ali-Shtayeh et al. 2022, Ali-Shtayeh et al. 2022a, Ali-Shtayeh et al. 2022b, Gedeon and Qumsiyeh 2023, Qumsiyeh and Al-Sheikh 2023, Al-Sheikh et al. 2024, Ali-Shtayeh et al. 2025a, Ali-Shtayeh et al. 2025b, Ali-Shtayeh et al. 2025c). Advances in plant systematics, the increasing availability of digital herbarium resources and the widespread use of global taxonomic resources, such as Plants of the World Online (POWO) (POWO 2023), the World Flora Online (WFO) and the International Plant Names Index (**IPNI**) (IPNI 2023), have further necessitated the re-assessment of earlier published checklists. Given that several plant taxa have been newly recorded or taxonomically re-assessed, while others have been excluded or reclassified at the species or subspecies levels, the preparation of an updated checklist reflecting current floristic and taxonomic knowledge is needed.

In addition to taxonomic progress, escalating pressures from habitat degradation, land‑use change and climate stress across the eastern Mediterranean have intensified the need for robust, conservation-orientated floristic baselines. According to the recent national and regional Red List initiatives, a substantial proportion of the Mediterranean flora faces increased extinction risk, mainly amongst habitat specialists and strict endemics. For the Palestinian West Bank, updated IUCN‑based assessments (Ali-Shtayeh et al. 2022a, Ali-Shtayeh et al. 2025a) underline the urgency of clearly documenting native species diversity, patterns of endemism and local extinctions.

The current provision resembles a second updated checklist of the native vascular flora of the West Bank of the State of Palestine, focusing exclusively on indigenous taxa of this area. Extreme restrictions on field surveys, specimen verification and data access forced by the ongoing war on Gaza since 2023 have rendered a comprehensive and reliable floristic update for that area currently unfeasible.

Unlike previous national floristic syntheses, the present study integrates newly-published floristic records, updated digital herbarium and biodiversity database evidence, harmonised taxonomy based on current international standards and recently completed national IUCN conservation assessments within a unified framework, thereby providing the most up-to-date and methodologically standardised checklist of the native vascular flora of the Palestinian West Bank. By integrating critically revised taxonomy, harmonised nomenclature, recent floristic records and conservation status information, the current checklist aims at: (i) serving as a standardised taxonomic reference for botanical and ecological research; (ii) facilitating comparison with other current Mediterranean floras and (iii) supporting evidence-based conservation planning and biodiversity monitoring at national and regional levels.

## Material and methods

The present checklist is based on the most recent annotated checklist of the native vascular flora for the State of Palestine (Ali-Shtayeh et al. 2022), which served as the baseline reference for taxonomic content, distributional data and ecological attributes. A comprehensive critical revision of species and infraspecific taxa was undertaken, incorporating new floristic records, taxonomic updates and nomenclatural changes published since the previous checklist.

Taxonomic nomenclature was verified and updated through consultation of the World Flora Online (WFO, www.worldfloraonline.org), complemented by original protologues and major international nomenclatural databases, particularly the International Plant Names Index (IPNI 2023, onwards) and the World Flora Online Plant List (WFO 2018, onwards, formerly *The Plant List*).

The systematic arrangement and taxonomic circumscription of plant families follow the (I.P.P.G. 2016) for ferns and lycophytes, Christenhusz et al. (2011) for gymnosperms and APG (2016) for angiosperms. Author citations for plant names were standardised according to IPNI (IPNI 2023, onwards) and the World Flora Online.

Occurrence records were compiled from published literature, herbarium specimens and field observations and biodiversity databases, such as BioGIS (BioGIS 2025) and the Global Biodiversity Information Facility (GBIF) (GBIF 2025). All records were critically evaluated to confirm taxonomic identity and geographic reliability. For each taxon, concise notes on taxonomy, nomenclature, distribution and conservation status are provided in Suppl. material 1.

The geographical distribution of the West Bank, within the State of Palestine, is classified into six phytogeographical districts: the Dead Sea Valley (DSV), Nablus Mountains (NM), Nablus Wilderness (NW), Jerusalem and Hebron Mountains (JHM), Jerusalem and Hebron Wilderness (JHW) and the Lower Jordan Valley (LJoV). These districts reflect the principal biogeographic, climatic and topographic gradients within the present study.

The conservation status of the plant taxa was assigned using the IUCN Red List categories at different assessment levels, where available: Palestine Red List (PAL RL) (Ali-Shtayeh et al. 2025a, Ali-Shtayeh et al. 2025c), Jordan Red List (Jo RL) (Taifour and El-Oqlah 2015, Taifour 2017), Global Red List (Glob RL) (IUCN 2025) and Historic Palestine (HPAL) (Sapir et al. 2003). The categories applied are: Least Concern (LC), Near Threatened (NT), Vulnerable (VU), Endangered (EN), Critically Endangered (CR), Extinct or Possibly Extinct (EX) and Data Deficient (DD).

Data on endemic taxa were derived from the updated Palestinian endemic plant inventory (Ali-Shtayeh et al. 2022) and the continuously updated World Flora Online (WFO 2018). Endemism was coded using the following categories: Endemic to Palestine (EP); Palestine and Israel (EPI); Palestine and Jordan (EPJ); Palestine, Israel and Jordan (EPIJ); Palestine, Jordan and Syria (EPSJ); Palestine, Israel and Syria (EPIS); Palestine, Israel, Jordan and Syria (EPIJS); and Palestine, Israel, Jordan, Syria and adjacent regions (EPIJSE).

Habitat preferences for each taxon were classified using the following categories (Zohary 1973): Batha (A) [a Mediterranean dwarf shrubland vegetation type dominated by low, drought-adapted aromatic shrubs and grasses]; Desert (B); Humid habitats (C); Sand (D); Disturbed habitats (E); Mediterranean maquis and forest (F); Shrub-steppes (G); Hard rock outcrops (H); Cultivated areas/weeds (I); Saline habitats (J); Nutrient-rich soils (K); Ruderal habitats (L); Mediterranean strand (M) [a coastal habitat above the high-tide line with sparse salt-tolerant vegetation adapted to sun and salt spray]; Tragacanth shrub vegetation (Oro-Mediterranean) (N); Shady rocks (O); Mediterranean grasslands (P); and Walls (Q) [vegetation growing on vertical man-made structures such as stones or brick walls that provide microhabitats for specialised species].

Chorological affinities (chorotypes (ChT)) were assigned following the widely used biogeographical schemes, including Irano-Turanian (IT), Mediterranean (M), Euro-Siberian (ES), Saharo-Arabian (SA), Sudanian (SUD), Tropical (T), American (A), Australian (AU), African (AFR) and Pluriregional Boreo-Tropical (PT). Growth forms (GF) were classified as: Annual (A), Phanerophytic shrub (PhS), Phanerophytic tree (PhT), Hemicryptophyte (H), Geophyte (G), Vine (V), Biennial (B), Helophyte (HE) and Sub-shrub/Chamaephyte (C).

Pollination systems (Poll) were classified as: animal-pollinated (Z), wind-pollinated (W), mixed wind and animal pollination (M) and water pollination (H). Climatic zones were defined as Mediterranean (MED), Transition Zone (TZ) and Semi- to Extreme Desert (D).

## Results

The updated checklist of the native vascular flora of the Palestinian West Bank (Suppl. material 1) comprises a total of 1,710 taxa, including 1,668 species and 42 subspecies and varieties distributed amongst 652 genera and 102 families. Pteredophytes are represented by 10 taxa belonging to three families and five genera, gymnosperms are represented by five taxa belonging to three genera and three families, whereas angiosperms account for 1,695 taxa grouped into 640 genera and 96 families.

**Taxonomic composition**

The floristic composition is dominated by a limited number of species-rich families (≥ 15 taxa), which together represent a substantial proportion of the native flora (Table 1). The most represented families are Fabaceae (198 taxa; 11.58%), Asteraceae (184 taxa; 10.82%), Poaceae (161 taxa; 9.42%), Caryophyllaceae (82 taxa; 4.80%), Brassicaceae (80 taxa; 4.68%) and Lamiaceae (80 taxa; 4.68%). At the generic level, the most species-rich genera (≥ 10 taxa) are *Trifolium*, *Allium*, *Silene*, *Medicago*, *Astragalus* and *Salvia* (Table 2), reflecting the strong representation of Mediterranean and Irano-Turanian floristic elements in the regional flora.

**Extinct and possibly extinct taxa**

In addition to the 1,710 taxa recorded, 29 taxa have not been confirmed in recent decades and are, therefore, considered extinct or possibly extinct within the Palestinian West Bank (Suppl. material 2). These taxa include species historically associated with wetlands, grasslands, Mediterranean woodlands and desert margins, such as *Sambucus nigra*, *Aegilops vavilovii*, *Leptadenia pyrotechnica*, *Orchis italica* and *Limonium sinuatum*. Amongst these is the subendemic species *Sporobolus minuartioides*, which has not been recorded in recent surveys and is, therefore, considered locally extinct. The apparent loss of these taxa reflects the ongoing habitat degradation, the land-use change and hydrological alteration across the region.

**Conservation status**

**Figure 1.**
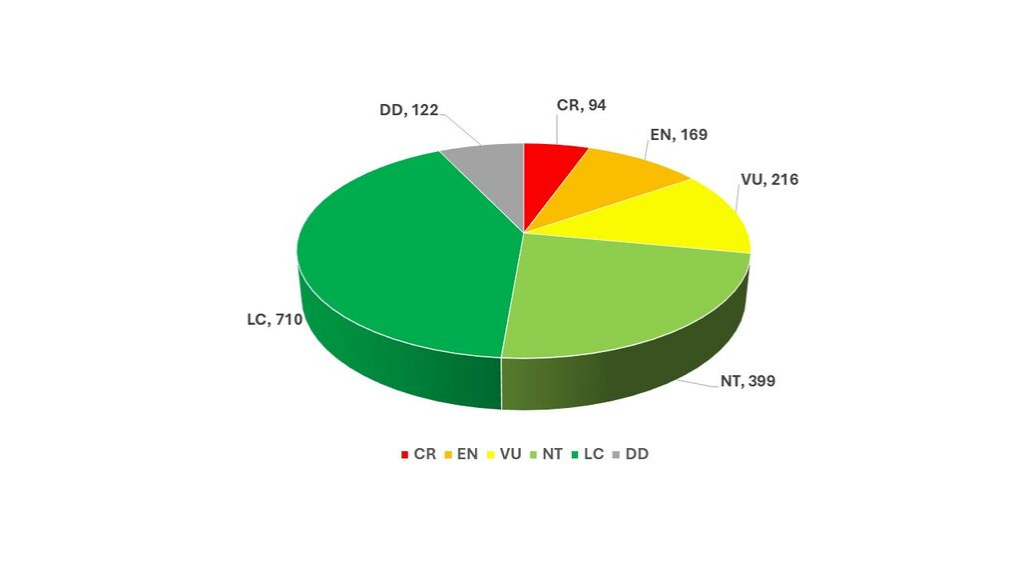
Distribution of plant taxa according to the IUCN Criteria.

IUCN Red List assessments for the native vascular flora of the Palestinian West Bank of the State of Palestine indicate that 482 taxa (28.2%) fall within the threatened categories, including 94 Critically Endangered (CR), 169 Endangered (EN) and 216 Vulnerable (VU) taxa (Fig. 1). In addition, 399 taxa are classified as Near Threatened (NT), 710 taxa as Least Concern (LC) and 122 taxa as Data Deficient (DD).

**Figure 2.**
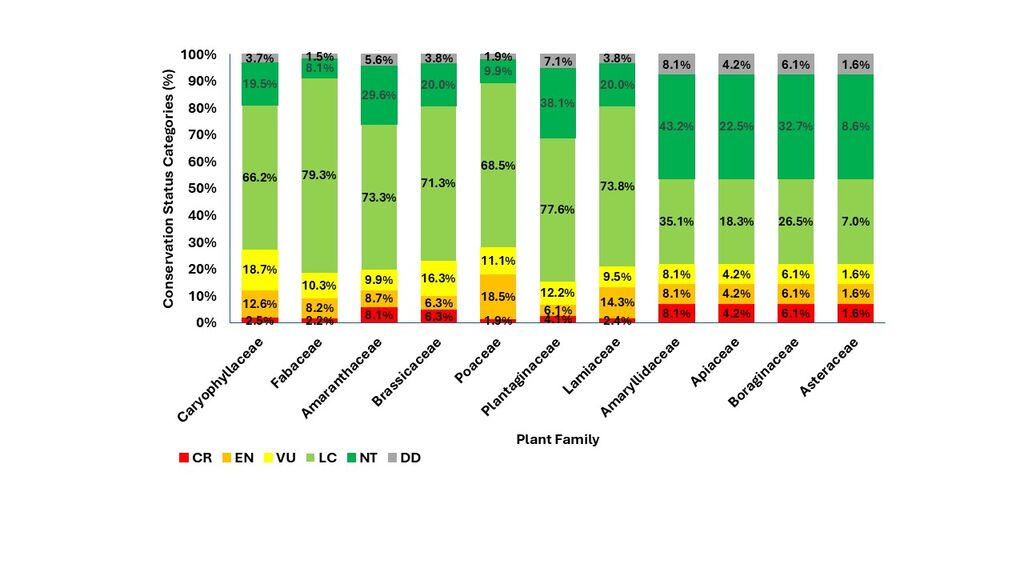
Distribution of taxa, based on their conservation status in the most represented plant families.

These findings highlight substanial conservation concern affecting a large proportion of the native flora, particularly in habitats subject to intensive anthropogenic pressure. The distribution of threatened categories (CR, EN and VU) varies considerably between families, with the following families (≥ 35 taxa) having the highest percentages of threatened taxa: Caryophyllaceae (34.1%, 28 taxa), Fabaceae (33.8%, 38), Amaranthaceae (31.5%, 17) and Brassicaceae (28.8%, 23) (Fig. 2). Families with the highest percentage of Critically Endangered CR taxa include: Amaranthaceae and Amaryllidaceae (8.1%), Brassicaceae (6.3%) and Boraginaceae (6.1%).

**Endemic flora**

A total of 151 taxa are considered endemic or sub-endemic to Palestine, belonging to 81 genera and 34 families (Table 3). Amongst these, two taxa — Iris lortetii var. samariae (Iridaceae) and *Ferula samariae* (Apiaceae) — are strictly endemic to Palestine. The families with the highest representation of endemic taxa are Asteraceae (32 taxa), Lamiaceae (13), Fabaceae (12), Amaryllidaceae (12), Boraginaceae (8) and Iridaceae (8). The most represented endemic taxa at the generic level include: *Allium*, *Trifolium*, *Centaurea*, *Onopordum*, *Salvia*, *Verbascum* and *Iris* (Table 4).

**Figure 3.**
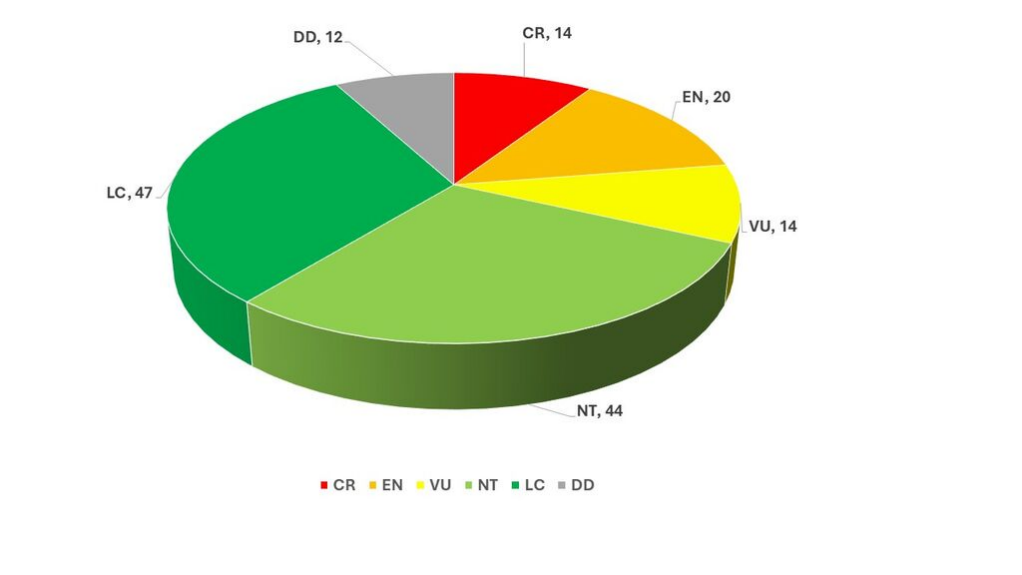
Distribution of IUCN categories amongst endemic plants.

The distribution of IUCN categories amongst endemic taxa reveals a strong concentration of endemics within threatened categories, reflecting restricted geographic ranges, habitat specificity and vulnerability to environmental change (Fig. 3).

**Figure 4.**
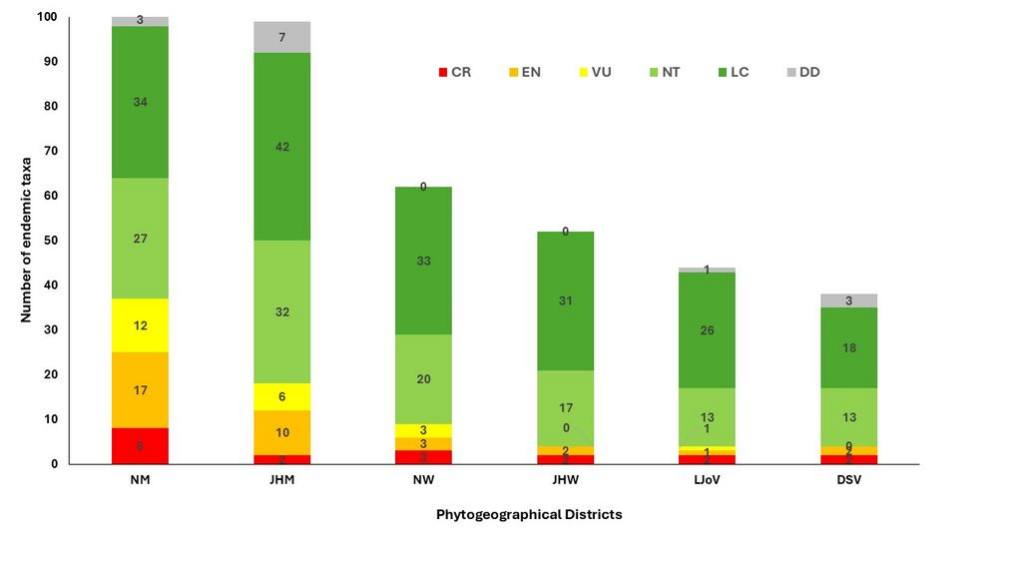
Number of Palestinian endemic species and subspecies according to the Palestinian Red Listing and according to the IUCN Criteria in the six phytogeographical districts.

Marked spatial variation occurs in the conservation status of endemic taxa across the six phytogeographical districts (Fig. 4). Threatened endemic (CR, EN and VU) species are irregularly distributed, designating certain districts of significant importance for targeted conservation action. Critically Endangered endemic taxa are most concentrated in the Nablus Mountains (NM), represented by eight species, whereas other districts support fewer highly threatened species. Endandered (EN) endemics display a similar pattern, with NM and JHM hosting 17 and 10 species, respectively. Other districts harbour comparatively few EN endemics, suggesting either a reduced number of endemic species or a moderately minimal extinction risk in DSV, LJoV, JHW and NW. This distribution pattern features NM as a critical hotspot for highly threatened endemic taxa, likely reflecting both pronounced habitat specificity and substantial anthropogenic pressures. This trend is supported by the VU category, where the NM and JHM hosts 12 and six taxa, respectively. Although DSV and JHW harbour VU endemics, their contribution of moderately threatened species remains restricted.

NT and LC endemics dominate in all phytogeographical districts, with the highest numbers of NT taxa occurring in JHM (32) and NM (27). LC endemics are most dominant in JHM (42 taxa), NM (34) and NW (33). The current results indicate that mountainous regions (NM and JHM) support not only highly threatened endemic flora, but also a substantial pool of flora that may become threatened under changing conditions.

**Phytogeographical distribution**

**Figure 5.**
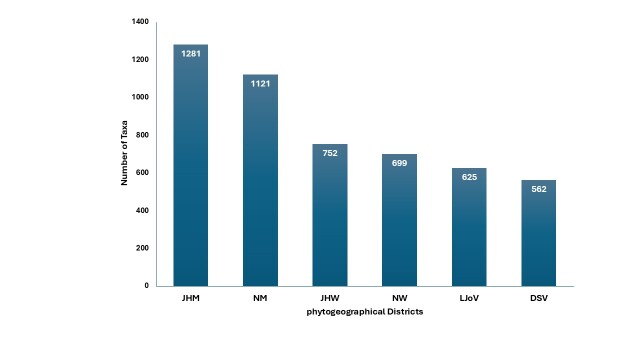
Distribution of plant taxa according to phytogeographical districts.

The spatial distribution of vascular plants across the phytogeographical districts of the Palestinian West Bank is highly uneven (Fig. 5). The western mountainous districts, which are characterised by relatively high precipitation and Mediterranean climatic conditions, exhibit the greatest floristic diversity. The Jerusalem and Hebron Mountains (JHM) supports 1,235 taxa, followed by the Nablus Mountains (NM) with 1,126 taxa. In contrast, eastern districts, dominated by semi-arid to arid climatic regimes, sustain comparatively fewer taxa, including the Nablus Wilderness (699 taxa) and Jerusalem and Hebron Wilderness (752 taxa). Desert steppe and desert regions — namely the Dead Sea Valley (562 taxa) and the Lower Jordan Valley (625 taxa) — harbour the lowest species richness, reflecting both climatic limitations and reduced habitat heterogeneity.

## Discussion

**Floristic diversity and taxonomic revision in a Mediterranean context**

The updated checklist documents 1,710 native taxa in the Palestinian West Bank, confirming the area as a major centre of plant diversity within the Eastern Mediterranean despite its relatively small geographic extent and pronounced environmental gradients (Suppl. material 1). Differences between the present inventory and earlier compilations should be interpreted primarily as a consequence of improved taxonomic resolution, nomenclatural harmonisation, exclusion of doubtful records and stricter delimitation of native status rather than as evidence of changes in the actual biodiversity. The incorporation of newly-published floristic records, updated herbarium and biodiversity database evidence and recent conservation assessments has enabled a more accurate and standardised representation of the regional flora.

Comparable revisions have been reported across the Mediterranean Basin, where national plant inventories have undergone quantitative adjustments following the adoption of unified taxonomic frameworks and standardised inclusion criteria. Examples include the updated Italian checklist and the continuously revised Flora of Greece, in which changes in species numbers were largely driven by taxonomic re-circumscription, synonymisation and improved data validation rather than genuine biodiversity dynamics (Dimopoulos et al. 2016, Bartolucci et al. 2024). Both checklists documented changes primarily driven by taxonomic circumscription, exclusion of doubtful records and refinement of native status. However, the present study extends beyond a routine checklist update by integrating taxonomy, distributional evidence and conservation assessments within a unified methodological framework, representing the first synthesis of this type for the Palestinian West Bank. The transition toward dynamic, periodically updated floristic inventories is increasingly recognised as best practice in the Mediterranean biodiversity research because it enhances taxonomic consistency, facilitates cross-regional comparisons and supports long-term biodiversity monitoring. By aligning Palestinian floristic knowledge with contemporary Mediterranean standards, the present checklist provides the most up-to-date, conservation-relevant floristic baseline available for the area.

The present checklist builds upon, but substantially extends, the annotated floristic synthesis of Ali-Shtayeh et al. (2022), which provided an important baseline for the vascular flora of the State of Palestine. While the earlier study focused primarily on floristic composition and diversity patterns across both the West Bank and Gaza Strip, the current work introduces several significant advancements. First, it incorporates newly-published distributional records and taxonomic revisions that have appeared since 2022, together with additional herbarium and database evidence that was not previously synthesised. Second, the present checklist applies a more rigorous and updated taxonomic harmonisation using the latest international nomenclatural resources, resulting in refined species circumscription and improved delimitation of native status. Third and most importantly, the study explicitly integrates recently completed national IUCN Red List assessments (Ali-Shtayeh et al. 2025a), providing a comprehensive conservation framework that was not available at the time of the earlier publication. In addition, the present work focuses specifically on the Palestinian West Bank, allowing a more detailed phytogeographical and conservation analysis at subregional scale, including the documentation of nationally extinct or possibly extinct taxa. These methodological and data-driven advances collectively justify the preparation of the current updated checklist and demonstrate that it represents substantive scientific progress rather than a simple reiteration of previous inventories.

**Taxonomic composition and floristic structure**

The dominance of certain plant families (Fabaceae, Asteraceae, Poaceae, Caryophyllaceae, Brassicaceae, Lamiaceae and Apiaceae), reflects the same patterns described from other Mediterranean and eastern Mediterranean floras, such as Italy (Bartolucci et al. 2024), Greece (Dimopoulos et al. 2016) and Cyprus (Hand et al. 2011). These families comprise taxa characterised by ecological flexibility, diverse life forms and effective reproductive strategies that enable persistence under seasonal drought, grazing and long-standing human land use. At the generic level, the dominance of *Trifolium*, *Allium*, *Silene*, *Medicago*, *Astragalus* and *Salvia* reflects the combined influence of Mediterranean grassland systems and Irano-Turanian steppe elements, a pattern consistent with floristic syntheses from the broader Levant and Aegean regions. Such compositional patterns support classifying the Palestinian West Bank as a transitional floristic zone influenced by multiple phytogeographical regions.

**Phytogeographical gradients and spatial distribution**

Floristic richness in the Palestinian West Bank is strongly structured along west–east environmental gradients. Mountainous districts with Mediterranean climatic influence host the highest species richness, paralleling distribution patterns observed in the mountainous regions of Greece and southern Italy (Dimopoulos et al. 2016, Bartolucci et al. 2024). In contrast, eastern wilderness districts and the Jordan Valley support fewer taxa, but include a higher proportion of Saharo-Arabian and Sudanian elements, contributing disproportionately to regional biogeographical diversity. Similar contrasts between species-rich Mediterranean zones and floristically distinctive arid regions are well documented in other Mediterranean checklists, including Cyprus (Hand et al. 2011), reinforcing the need to evaluate floristic value beyond species counts alone. This contrast between species-rich Mediterranean zones and floristically distinctive arid regions underscores the importance of evaluating biodiversity value not only in terms of species richness, but also in terms of ecological uniqueness and biogeographical representation.

**Endemism and regional biogeographical significance**

The documentation of 151 endemic and sub-endemic taxa highlights the Palestinian West Bank’s as an important component of the Levantine centre of plant endemism. Although strict national endemics are relatively few, the high percentage of taxa shared with neighbouring countries reflects historical biogeographical connectivity and common evolutionary trajectories across the eastern Mediterranean (Zohary 1973, Takhtajan 1986, Médail and Diadema 2009). Comparable patterns of narrow endemism embedded within wider regional distributions have been reported in Italy and Greece, where endemic taxa are frequently concentrated in specific habitats or topographic units rather than confined to political borders (Crisp et al. 2002, Thompson et al. 2005, Dimopoulos et al. 2016, Bartolucci et al. 2024).

The representation of endemic taxa in Asteraceae, Lamiaceae, Fabaceae, Amaryllidaceae and Iridaceae families in the Palestinian West Bank is similar to family-level patterns reported across Mediterranean Plants (Taifour and El-Oqlah 2015, Dimopoulos et al. 2016, Bartolucci et al. 2024). Threatened taxa (CR, EN and VU) are extremely concentrated in mountainous districts (NM and JHM), thus identifying these districts as critical hotspots of endemic plant diversity and conservation concern, which reflects topographic complexity, habitat specialisation and sustained anthropogenic pressure (Médail and Diadema 2009). However, as demonstrated by Orsenigo et al. (2018), endemic richness alone does not necessarily guarantee effective protection, especially when anthropogenic pressures remain high. The dominance of Near Threatened and Least Concern endemic taxa in these districts may, therefore, represent a reservoir of species potentially vulnerable to rapid status deterioration under increasing environmental stress, reinforcing the importance of proactive conservation strategies.

From a conservation perspective, endemic taxa represent particularly important elements because their persistence depends largely on regional or national conservation actions. Orsenigo et al. (2018) emphasised that taxa under full national responsibility constitute key priorities for biodiversity management, as their extinction would represent an irreversible biodiversity erosion. Although Palestine hosts relatively few strictly national endemics compared with larger Mediterranean countries, the presence of narrow-range taxa and sub-endemics still confers high conservation value at the regional scale.

**Conservation status and extinction signals**

The IUCN assessment of the floristic taxa in the Palestinian West Bank indicates that 28.2% of the native flora fall within threatened categories (CR, EN and VU). This percentage is almost comparable to or exceeding those reported in other Mediterranean biodiversity hotspots, where about 20-28% of the assessed flora are often reported as threatened with extinction risk, strongly associated with anthropogenic habitat modification, land-use change and coastal or lowland development pressures (Dimopoulos et al. 2016, Orsenigo et al. 2021). Recent national assessments in Italy and Greece similarly demonstrate that a substantial fraction of native floras faces an elevated extinction risk, particularly amongst habitat specialists and narrow endemics (Dimopoulos et al. 2016, Orsenigo et al. 2021, Bartolucci et al. 2024). Of particular concern is the documentation of 29 extinct or possibly extinct taxa at the national level. Many of these taxa were historically associated with wetlands, traditional agro-ecosystems or lightly grazed grasslands, which have undergone substantial transformation due to agricultural intensification, urban expansion and hydrological alteration. Comparable losses of wetland and lowland taxa have been reported in Mediterranean countries where hydrological alteration and land-use intensification have occurred over the past century, including Cyprus and southern Italy (Hand et al. 2011, Bartolucci et al. 2024). Similarly, Orsenigo et al. (2018) demonstrated that residential development and tourism represent a dominant driver of extinction risk in Mediterranean endemic plants. The parallel patterns observed in the Palestinian West Bank, therefore, reflect broader regional processes of biodiversity erosion associated with socio-economic development. The identification of extinction signals also highlights the importance of continuous monitoring and re-assessment of species populations. In Mediterranean contexts, extinction often results from gradual population decline rather than abrupt disappearance, meaning that early detection and targeted management interventions are essential to prevent further biodiversity loss.

In addition to these ecological drivers, socio-environmental pressures associated with the ongoing conflict in the region may also contribute to habitat degradation and biodiversity decline. According to the IUCN Threat Classification Scheme, war, civil unrest and military activities are recognised as potential threats to biodiversity (6.2 War, Civil unrest and military exercises). In the Palestinian West Bank, military activities and land-use changes associated with the expansion of Israeli settlements and related infrastructure have contributed to habitat fragmentation and disturbance in some areas. Furthermore, restrictions on land access and limitations on traditional agricultural and pastoral management practices may disrupt long-established Mediterranean agroecosystem dynamics that historically maintained habitat heterogeneity and supported high plant diversity. The decline of traditional grazing regimes and low-intensity cultivation systems may, therefore, contribute to the local decline or disappearance of plant populations, particularly amongst habitat specialists and narrowly distributed taxa.

**Methodological robustness and broader relevance**

The major strength of the current plant checklist lies in the standardisation of taxonomy, different data sources and precise conservation coding, dependent on continuing Mediterranean checklist initiatives. With the adoption of internationally accepted taxonomic supports and incorporation of the recent floristic evidence, the checklist functions as a living baseline, comparable in scope and structure to other leading Mediterranean floras, such as those of Italy, Greece and Cyprus (Hand et al. 2011, Dimopoulos et al. 2016, Bartolucci et al. 2024). This organisation facilitates cross-regional comparisons and strengthens the checklist’s value for biodiversity monitoring, biogeographical analysis and plant conservation planning. Supplementary Material S1 (Suppl. material 1) provides a comprehensive floristic backbone, while Supplementary Material S2 (Suppl. material 2) offers a rare, explicitly documented account of local plant extinctions, substantially increasing the checklist’s scientific and conservation value.

## Conclusions

The revised checklist provides the most current and standardised synthesis of the native vascular flora of the Palestinian West Bank and offers a robust baseline for biodiversity monitoring and conservation planning. The substantial proportion of threatened taxa, together with the documented local extinction and the concentration of endemic diversity in specific geographic districts, underscores the critical need for immediate and targeted conservation actions. Consistent with Mediterranean conservation analyses, including those of Orsenigo et al. (2018), effective protection of endemic and threatened flora requires not only the designation of protected areas, but also the evaluation of their spatial effectiveness and management adequacy. Endemic-rich regions do not necessarily coincide with existing conservation networks and management interventions should, therefore, prioritise areas identified as biodiversity hotspots through floristic and conservation analyses.

Based on the findings of this study, several priority actions are recommended: (a) identification and formal protection of endemic-rich mountainous districts as national conservation priority areas; (b) long-term monitoring programmes for threatened and Data Deficient taxa, particularly narrow-range species vulnerable to habitat modification; (c) restoration and sustainable management of traditional agro-ecosystems and wetlands, which historically supported high plant diversity and now show strong extinction signals; (d) integration of updated floristic data into national biodiversity strategies and land-use planning, ensuring that conservation priorities are informed by current scientific evidence and (e) development of species-specific conservation action plans for critically endangered taxa, consistent with international best practices for plant conservation.

By aligning regional floristic knowledge with contemporary Mediterranean conservation frameworks, this study contributes essential information for safeguarding plant diversity in the Palestinian West Bank and supports broader efforts to protect biodiversity across the Levant.

## Supplementary Material

75549EFA-0254-58E6-9DBC-3711621F22CB 10.3897/BDJ.14.e188380.suppl1 Supplementary material 1 The updated checklist of the native vascular flora of the Palestinian West Bank Data type Occurrences Brief description This supplementary dataset provides a comprehensive and standardised checklist of native vascular plant taxa recorded from the Palestinian West Bank. It includes accepted scientific names with relevant synonyms, family assignment, English and Arabic vernacular names, life forms, pollination systems, chorological elements, habitat preferences and associated climatic regions. Conservation-related information is provided where available, including global IUCN Red List status, national conservation status, endemism and rarity. Herbarium voucher details and bibliographic references are included for taxonomic verification. The dataset serves as a baseline reference for floristic research, biodiversity assessment and conservation planning in the Palestinian West Bank. File: oo_1557742.xlsx https://binary.pensoft.net/file/1557742 M. S. Ali-Shtayeh*, Salam Y. AbuZaitoun, Rana M. Jamous

82D0735A-F734-5837-9909-6760D11FA218 10.3897/BDJ.14.e188380.suppl2 Supplementary material 2 List of Palestinian extinct vascular taxa Data type Occurrences Brief description Checklist of vascular plant taxa considered extinct at the national level in Palestine, arranged by family. Accepted scientific names with authorship are provided, with principal taxonomic synonyms indicated where relevant. The list is based on evaluation of historical records, herbarium evidence and recent field surveys and supports taxonomic transparency and conservation assessments. File: oo_1536573.docx https://binary.pensoft.net/file/1536573 Ali-Shtayeh MS, Abuzairoun SY, Jamous RM

## Figures and Tables

**Table 1.** Most represented families (≥ 15 taxa) of the Palestinian West Bank native vascular flora.

Family Name	Taxa (%)	Genera (%)
Fabaceae	198 (11.58)	37 (6.67)
Asteraceae	184 (10.82)	82 (12.58)
Poaceae	161 (9.42)	75 (11.50)
Caryophyllaceae	82 (4.80)	26 (3.99)
Brassicaceae	80 (4.68)	54 (8.28)
Lamiaceae	80 (4.68)	25 (3.83)
Apiaceae	71 (4.15)	39 (5.98)
Amaranthaceae	54 (3.16)	21 (3.22)
Boraginaceae	49 (2.87)	21 (3.22)
Plantaginaceae	42 (2.46)	10 (1.53)
Amaryllidaceae	37 (2.16)	4 (0.61)
Asparagaceae	31(1.81)	10 (1.53)
Rubiaceae	31 (1.81)	11 (1.69)
Orchidaceae	29 (1.70)	8 (1.23)
Cyperaceae	28 (1.64)	11 (1.69)
Ranunculacea	28 (1.64)	9 (1.38)
Caprifoliaceae	26 (1.52)	9 (1.38)
Papaveraceae	26 (1.52)	7 (1.07)
Convolvulaceae	24 (1.40)	3 (0.46)
Malvaceae	21 (1.23)	7 (1.07)
Geraniaceae	21 (1.23	2 (0.31)
Euphorbiaceae	21(1.17)	3 (0.46)
Polygonaceae	20(1.17)	4 (0.61)
Scrophulariaceae	20(1.17)	2 (0.31)
Cistaceae	15 (0.88)	3 (0.46)
Iridaceae	15 (0.88)	4 (0.61)
Orobanchaceae	15 (0.88)	5 (0.77)

**Table 2.** Most represented genera (≥ 15 taxa) of the Palestinian West Bank native vascular flora.

Genus	Taxa (%)	Family Name
* Trifolium *	35 (2.05)	Fabaceae
Allium	33 (1.93)	Amaryllidaceae
* Silene *	31 (1.81)	Caryophyllaceae
* Medicago *	23 (1.35)	Fabaceae
* Astragalus *	22 (1.29)	Fabaceae
* Salvia *	19 (1.11)	Lamiaceae
* Euphorbia *	17 (0.99)	Euphorbiaceae
* Bromus *	17 (0.99)	Poaceae
* Convolvulus *	15 (0.99)	Convolvulaceae
* Lathyrus *	15 (0.99)	Fabaceae
* Vicia *	15 (0.99)	Fabaceae
* Plantago *	15 (0.99)	Plantaginaceae
* Galium *	15 (0.99)	Rubiaceae

**Table 3.** Plant families represented in endemic taxa, with the number of species.

**Family**	**Species (%)**
** Asteraceae **	32 (21.19)
** Lamiaceae **	13 (8.61)
** Boraginaceae **	8 (5.30)
** Apiaceae **	6 (3.97)
** Fabaceae **	12 (7.95)
** Brassicaceae **	4 (2.65)
** Asparagaceae **	5 (3.31)
** Apocynaceae **	3 (1.99)
** Amaryllidaceae **	12 (7.95)
** Iridaceae **	8 (5.30)
** Scrophulariaceae **	7 (4.64)
** Caryophyllaceae **	5 (3.31)
** Tamaricaceae **	3 (1.99)
** Amaranthaceae **	3 (1.99)
** Plantaginaceae **	2 (1.32)
** Poaceae **	2 (1.32)
** Araceae **	2 (1.32)
** Caprifoliaceae **	2 (1.32)
** Rubiaceae **	4 (2.65)
** Campanulaceae **	4 (2.65)
** Colchicaceae **	3 (1.99)
** Dioscoreaceae **	1 (0.66)
** Acanthaceae **	1 (0.66)
** Convolvulaceae **	1 (0.66)
** Crassulaceae **	1 (0.66)
** Euphorbiaceae **	1 (0.66)
** Liliaceae **	1 (0.66)
** Malvaceae **	1 (0.66)
** Ranunculaceae **	1 (0.66)
** Resedaceae **	1 (0.66)
** Rhamnaceae **	1 (0.66)
** Zygophyllaceae **	1 (0.66)

**Table 4.** Most represented genera (≥ 3 taxa) of the Palestinian endemic vascular flora.

Genus	**Species (%)**	**Family Name**
* Allium *	11 (7.28)	Amaryllidaceae
* Trifolium *	7 (4.64)	Fabaceae
* Onopordum *	6 (3.97)	Asteraceae
* Anthemis *	5 (3.31)	Asteraceae
* Centaurea *	5 (3.31)	Asteraceae
* Iris *	5 (3.31)	Iridaceae
* Salvia *	5 (3.31)	Lamiaceae
* Verbascum *	5 (3.31)	Scrophulariaceae
Galium	4 (2.65)	Rubiaceae
* Silene *	4 (2.65)	Caryophyllaceae
* Campanula *	3 (1.99)	Campanulaceae
* Colchicum *	3 (1.99)	Colchicaceae
* Crocus *	3 (1.99)	Iridaceae
* Ornithogalum *	3 (1.99)	Asparagaceae
* Stachys *	3 (1.99)	Lamiaceae
* Trigonella *	3 (1.99)	Fabaceae
