# Supplementary material for: An updated checklist of the vascular flora native to the State of Palestine – West Bank
Source: Biodivers Data J. 2026 Mar 31;14:e188380. doi: 10.3897/BDJ.14.e188380 (PMC13058597; doi:10.3897/BDJ.14.e188380)
Supplement: Supplementary material 2 — List of Palestinian extinct vascular taxa [file bdj-14-e188380-s002.docx]

Supplementary Material S2: List of Palestinian extinct vascular taxa.

| **Scientific name (synonyms)** |
| --- |
| **Adoxaceae** |
| *Sambucus nigra* L. |
| **Alismataceae** |
| *Alisma lanceolatum* With. |
| **Apiaceae** |
| *Bunium ferulaceum* Sm. |
| *Bupleurum orientale* Snogerup |
| *Oenanthe prolifera* L. |
| *Scandix iberica* M.Bieb. |
| **Apocynaceae** |
| *Leptadenia pyrotechnica* (Forssk.) Decne. |
| **Asteraceae** |
| *Laphangium luteoalbum* (L.) Tzvelev (*Pseudognaphalium luteoalbum* (L.) Hilliard & B.L.Burtt) |
| **Boraginaceae** |
| *Alkanna orientalis* (L.) Boiss. |
| **Brassicaceae** |
| *Lobularia arabica* (Boiss.) Muschl. |
| **Cyperaceae** |
| *Cyperus jeminicus* Rottb |
| **Euphorbiaceae** |
| *Euphorbia forskaolii* J.Gay |
| **Fabaceae** |
| *Lotus ornithopodioides* L. |
| *Medicago murex* Willd. |
| Medicago tornata (L.) Mill. [Medicago italica (Mill.) Fiori] |
| *Tephrosia purpurea subsp. apollinea* (Delile) Hosni & El-Karemy [*Tephrosia apollinea* (Delile) Link] |
| **Juncaceae** |
| *Juncus sphaerocarpus* Nees |
| **Lamiaceae** |
| *Clinopodium barbatum* (P.H.Davis) Melnikov |
| *Lamium orientale* (Fisch. & C.A.Mey.) E.H.L.Krause [*Wiedemannia orientalis* Fisch. & C.A Mey.] |
| **Malvaceae** |
| *Alcea striata* (DC.) Alef. |
| **Orchidaceae** |
| *Orchis italica* Poir. |
| **Plantaginaceae** |
| *Globularia arabica* Jaub. & Spach |
| **Plumbaginaceae** |
| *Limonium sinuatum* (L.) Mill. |
| **Poaceae** |
| *Aegilops vavilovii* (Zhuk.) Chennav. |
| *Alopecurus alopecuroides* (L.) L.J.Gillespie, Cabi & Soreng *(Cornucopiae alopecuroides* L.) |
| *Avena fatua* L. |
| *Hemarthria altissima* (Poir.) Stapf & C.E.Hubb. |
| *Sporobolus borszczowii subsp. acuminatus* (Trin.) P.M.Peterson *(Crypsis acuminata* Trin.) |
| *Sporobolus minuartioides* (Bornm.) P.M.Peterson *(Crypsis minuartioides* (Bornm.) Mez) |
